# Use of synthetic adrenocorticotropic hormone in patients with IgA nephropathy

**DOI:** 10.1186/s12882-018-0915-4

**Published:** 2018-05-23

**Authors:** Bhanu Prasad, Shelley Giebel, Michelle C. E. McCarron, Nelson Leung

**Affiliations:** 1 Section of Nephrology, Department of Medicine, Regina General Hospital, 1440, 14th Avenue, Regina, SK S4P 0W5 Canada; 2 Faculty of Nursing, Research and Innovation Centre, University of Regina, Room 508, 3737, Wascana Parkway, Regina, SK S4S 0A2 Canada; 3 Research and Performance Support, Regina Qu’Appelle Health Region, 2180-23rd Avenue, Regina, SK S4S 0A5 Canada; 4 Nephrology and Hypertension, Mayo Clinic, 200 First Street, Minnesota, Rochester, MN 55905 USA

**Keywords:** IgA nephropathy, Adrenocorticotropic hormone, Proteinuria, Prednisone

## Abstract

**Background:**

Synthetic adrenocorticotropic hormone (ACTH) has been demonstrated to be effective in patients with membranous nephropathy, minimal change disease and some histological subtypes of focal segmental glomerulosclerosis. Its clinical impact in patients with IgA nephropathy is currently unclear.

**Case presentation:**

In this report, we describe the clinical use of ACTH in patients with IgA nephropathy. Three female patients (24–44 years) with overt proteinuria received intramuscular (IM) ACTH for varying time periods (8–14 months). Pre-treatment urine protein varied from 2.9 g/d to 4.3 g/d.

**Conclusions:**

There was complete remission in one patient on ACTH monotherapy and in the other two when prescribed as a steroid-sparing agent in combination with cyclophosphamide. All three had resolution in proteinuria to less than 1 g/d and maintained their GFR to baseline values. There were no reported side effects at a once a week dose. This study illustrates that ACTH is an effective agent that is well tolerated with minimal side effects and can be used as an alternative to prednisone in patients with IgA nephropathy.

## Background

Immunoglobulin A (IgA) nephropathy is the most common primary glomerulopathy in the developed world [1, 2]. The progression to end stage renal disease (ESRD) is variable, but there is evidence to suggest that accelerated decline in kidney function is associated with proteinuria > 1 g/d in a dose-dependent fashion [3–5].

Synthetic Adrenocorticotropic hormone (ACTH) has been used for treatment of minimal change disease, membranous nephropathy and focal segmental glomerulosclerosis (FSGS). However, there is a paucity of published data on the use of ACTH in patients with IgA nephropathy. In this paper, we present data from 3 females (aged 24–44 years) from southern Saskatchewan, Canada with biopsy-proven IgA nephropathy.

## Case descriptions

All patients were treated with ACTH (Synacthen Depot, Cosyntropin Zinc hydroxide suspension, Novartis Pharmaceuticals, Basel, Switzerland) starting at a dose of 1 mg per week; the frequency of the medication was adjusted based on the clinical response. All patients had negative anti nuclear antibody (ANA), anti neutrophilic cytoplasmic antibody (ANCA), and anti glomerular basement membrane (GBM). C3, C4, and erythrocyte sedimentation rate (ESR) were within the normal range. All of them were treated with diuretics, statins, maximal dose of angiotensin-converting-enzyme inhibitor, 1200 mg of omega 3 fatty acids and 4000 IU of vitamin D.

### Case 1

A 24-year-old female was initially referred for hypertension, microhematuria, and sub-nephrotic proteinuria of 2.8-g/d and serum creatinine of 92 μmol/L. She underwent a kidney biopsy, which showed six glomeruli, three of which were sclerotic. On immunofluorescence, there was IgA staining in the mesangium. Electron microscopy revealed dense immune-type deposits in the paramesangial space. The diagnosis was consistent with IgA nephropathy.

The patient’s urine protein had increased to 5.02 g/d at 21 months post-diagnosis. 3 months later, her proteinuria remained elevated at 4.6 g/d; Mycophenolate Mofetil (MMF) was introduced at a dose of 1000 mg bid, as the patient expressed interest in trialing a non-prednisone-based immunosuppression with the fewest cutaneous side effects. Ten months post MMF, there was no significant improvement in her proteinuria to 2.78 g/d and she discontinued the medication at that point. She was without any immunosuppressant for 12 months. Her urine protein remained at 2.57 g/d with glomerular filtration rate (GFR) > 60 mls/minute and she agreed to be initiated on 1 mg/kg body weight of prednisone, to be tapered by 0.2 mg/kg every month. Within a month she had mild cushingoid features (acne, facial hair and weight gain), felt unwell, and her urine protein increased to 2.9 g/day. At this juncture, she was transitioned to IM ACTH 1 mg per week. Immediately after its introduction, the urine protein consistently improved from 2.90 g/d to 1.13 g/d, 0.78 g/day and 0.59 g/d. ACTH was discontinued at 11 months post initiation when her urine protein decreased to 0.46 g/d. At 10 months post discontinuation of ACTH, her urine protein was 0.92 g/d and her GFR remained > 60 mls/minute.

### Case 2

A 44-year-old female was referred for microhematuria, overt proteinuria and hypertension. Her urine protein was 3.04 g/d with creatinine of 99 μmol/L and GFR of 53 mls/min/1.73^m2^. She underwent a kidney biopsy, which showed 43 glomeruli, 20 of which were sclerotic. Four of the glomeruli showed FSGS with adhesions and fibrosis of the Bowman’s capsule. On immunofluorescence, there was 3+ IgA (diffuse, granular mesangial) staining. The diagnosis was consistent with IgA nephropathy. She received 3 doses of intravenous (IV) methyl prednisolone; IV cyclophosphamide was initiated at a dose of 1 g every 4 weeks for 6 doses and oral prednisone at a dose of 100 mg once a day (1 mg/kg body weight) was initiated, to be tapered at 0.2 mg/kg body weight per month. Despite receiving monthly IV cyclophosphamide and tapering doses of prednisone, her serum creatinine increased from 99 to 138 μmol/L, and there was no improvement with her urine protein (4.10 g/d). At that point, oral prednisone was discontinued and she was started on IM ACTH at 1 mg/week but continued monthly cyclophosphamide for a total of 6 doses. Four months after initiating ACTH, her urine protein had decreased to 1.07 g/day. Six months post initiation; the frequency of ACTH was decreased to 1 mg every other week. By 10 months post initiation, her urine protein had further decreased, to 0.88 g/day. At 14 months post initiation, her urine protein was 0.79 g/d with urea of 9.8 and creatinine of 138 μmol/L. At this point, the frequency of ACTH injections was decreased to 1 mg/month.

### Case 3

A 41-year-old female was initially reviewed with peripheral edema, overt proteinuria, microhematuria, and GFR > 60 mls/min/1.73^m2^. She underwent a kidney biopsy, which revealed 18 glomeruli, none of which were globally sclerotic. No mesangial, subendothelial or subepithelial deposits were observed. On immunofluorescence, there was 3 + IgA and C3 (diffuse fine granular mesangial and capillary wall) staining. Focal subepithelial and paramesangial dense immune type deposits were seen on electron microscopy. Based on the staining, the diagnosis was consistent with IgA nephropathy. Her admitting creatinine was 77 μmol/L; ACR was 447.3 mg/mmol and on quantification was 8.0 g/d. She received IV methylprednisone1 g/d for 3 days, followed by the Manno regime and a request was made to seek approval for ACTH from her insurance company [6]. Three weeks later, prednisone was discontinued due to weight gain, edema and acne and she was started on 1 mg ACTH IM per week. At 2 months post ACTH initiation, her urine protein was found to have elevated to 5.5 g/d, at which point the frequency of ACTH was increased to 1 mg twice per week. At 3 months post initiation, her urine protein had elevated further, to 6.09 g/d. Due to the lack of improvement on ACTH, it was decided to offer her IV cyclophosphamide at a dose of 1 g every 4 weeks for 6 months. Following its introduction, her urine protein sequentially improved each month, to 1.36 g/d, 0.77 g/d and 0.30 g/d. As her condition improved, the frequency of ACTH was reduced to every other week and then subsequently discontinued after 2 months. Four months later, she remains in remission.

## Discussion

In this manuscript, we demonstrate clinical use of IM ACTH in patients with IgA nephropathy. Cases 1 and 2 both showed close to 50% glomerular scarring at the time of biopsy, along with nephrotic range proteinuria but GFR >60mls/min/1.73^2^. Case 3 had typical signs and symptoms of nephrotic syndrome. All 3 were hypertensive and Case 2 had GFR of 53 mls/min/1.73^2^ at baseline. All three, if left untreated, had a high likelihood of progression to ESRD.

We used Synacthen depot/Cosyntropin zinc hydroxide—a synthetic, long-acting β1–24 corticotropin, which exhibits the same activity as natural ACTH with regards to its biological activities. The exact mechanism of action is debated with the stimulation of endogenous steroids potentially playing a role; however, recently published data suggest that it works on the melaonocortin receptors (MCRs) [7, 8]. MCRs are abundantly expressed on the glomerular podocytes, mesangial cells, endothelial and tubular epithelial cells and its stimulation has been demonstrated to reduce oxidative stress and improve glomerular morphology by decreasing apoptosis, injury and loss in the remnant kidney animal model [7, 9].

In one of the 3 patients, ACTH was used as a second line therapy (after MMF) and in the remainder; it was used as a steroid-sparing agent in conjunction with cyclophosphamide with excellent outcomes. The development and progression of IgA nephropathy involves multiple pathogenic mechanisms, and therefore the combination of ACTH and a second immunosuppressive agent that simultaneously targets different pathogenic pathways confer superior benefit. Typically, these patients would have been offered prednisone in the form of the Pozzi or Manno regimes [6, 10]. Its use would have exposed patients to the multitude of debilitating side effects of steroids.

## Conclusion

It is well documented that there is a dose response relationship of ACTH with improvement in proteinuria, but when the dose was increased to 1 mg twice per week in our cohort, patients invariably described side effects such as bloating, insomnia, a general feeling of unwell, and varying degrees of peripheral edema [11]. There is growing reluctance amongst physicians regarding the systemic side effects of prednisone, as evidenced by the use of steroid-free regimes in the treatment of primary GN [12]*.* This study illustrates that ACTH is a viable alternative to Prednisone (well tolerated with minimal side effects at lower doses) when added to Cyclophosphamide in patients with IgA nephropathy.

## Abbreviations

ACTH: Adrenocorticotrophic hormone
ANA: Anti-nuclear antibody
ANCA: Anti neutrophilic cytoplasmic antibody
anti GBM: Anti-glomerular basement membrane
MCR’s: Melanocortin receptors
MMF: Mycophenolate mofetil
